# The Seeking Mental Health Care model: prediction of help-seeking for depressive symptoms by stigma and mental illness representations

**DOI:** 10.1186/s12889-022-14937-5

**Published:** 2023-01-10

**Authors:** Thomas McLaren, Lina-Jolien Peter, Samuel Tomczyk, Holger Muehlan, Georg Schomerus, Silke Schmidt

**Affiliations:** 1 Department of Health and Prevention, Institute of Psychology, University of Greifswald, Robert-Blum Str. 13, 17489 Greifswald, Germany; 2 Department of Psychiatry and Psychotherapy, Medical Faculty, University Leipzig, Semmelweisstr. 10, 04103 Leipzig, Germany; 3 Department of Psychiatry and Psychotherapy, University of Leipzig Medical Center, Semmelweisstr. 10, 04103 Leipzig, Germany

**Keywords:** Depression, Help-seeking, Seeking mental health care model, Stigma, Path analysis, Healthcare use, Treatment experiences, Continuum beliefs, Biopsychosocial causal model, Self-efficacy

## Abstract

**Background:**

Only about half the people with depression seek professional health care services. To constitute the different predictors and associating variables of health care utilisation, we model the process and aim to test our hypothesised *Seeking Mental Health Care Model*. The model includes empirical influences on the help-seeking process to predict actual behaviour and incorporates superordinate (stigma, treatment experiences) as well as intermediate attitudinal variables (continuum and causal beliefs, depression literacy and self-efficacy).

**Method:**

All variables are examined in an online study (baseline, three- and six-month follow-up). The sample consisted of adults with depressive symptoms (PHQ-9 sum score ≥ 8), currently not receiving mental health care treatment. To examine the prediction of variables explaining help-seeking behaviour, a path model analysis was carried out (*lavaan package*, *software R*).

**Results:**

Altogether, 1368 participants (*M*_*age*_ = 42.38, *SD*_*age*_ = 15.22, 65.6% female) were included, 983 participating in at least one follow-up. Model fit was excellent (i.e., *RMSEA* = 0.059, *CFI* = 0.989), and the model confirmed most of the hypothesised predictions. Intermediary variables were significantly associated with stigma and experiences. Depression literacy (*ß* = .28), continuum beliefs (*ß* = .11) and openness to a balanced biopsychosocial causal model (*ß* = .21) significantly influenced self-identification (*R*^*2*^ = .35), which among the causal beliefs and self-efficacy influenced help-seeking intention (*R*^*2*^ = .10). Intention (*ß* = .40) prospectively predicted help-seeking behaviour (*R*^*2*^ = .16).

**Conclusion:**

The *Seeking Mental Health Care Model* provides an empirically validated conceptualisation of the help-seeking process of people with untreated depressive symptoms as a comprehensive approach considering internal influences. Implications and open questions are discussed, e.g., regarding differentiated assessment of self-efficacy, usefulness of continuum beliefs and causal beliefs in anti-stigma work, and replication of the model for other mental illnesses.

**Trial registration:**

German Clinical Trials Register: DRKS00023557. Registered 11 December 2020. World Health Organization, Universal Trial Number: U1111–1264-9954. Registered 16 February 2021.

**Supplementary Information:**

The online version contains supplementary material available at 10.1186/s12889-022-14937-5.

## Introduction

**Fig. 1:**
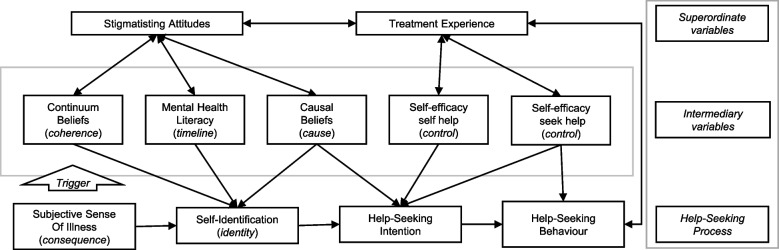
Seeking Mental Health Care Model. (Note: Superordinate and intermediary variables and their direct or indirect influences on the help-seeking process for mental health problems. In brackets: Illness representations from the *Common-Sense Model of Illness Regulation* assigned to the respective variables. Hypothesis on self-efficacy to seek help on behaviour was not postulated in the study protocol, but incorporated retrospectively due to previous findings and with the aim of analysing a comprehensive model)

Only around one in two people contact professional support services for their depressive symptoms, despite considering a broad range of services, including psychiatric or psychotherapeutic help, general practitioners, and counselling [1]. Even in regions with high healthcare density, there is a substantial proportion of people who do not seek professional help for their depressive symptoms [2]. The pathway to treatment is influenced by various individual, system, and disease factors [3], including availability and accessibility of services and structural stigma [4]. Even though all these factors are relevant, this work is focused on investigating personal attitudinal and cognitive variables to identify and overcome potential barriers for seeking mental healthcare [5] due to the importance of attitudinal barriers to mental health treatment for mild and moderate symptoms [6]. We provide a comprehensive *Seeking Mental Health Care Model* explaining the help-seeking process, as well as influences of superordinate and intermediate variables (see Fig. 1). In the following paragraphs, we will summarise key empirical findings on attitudinal and cognitive variables influencing professional help-seeking. We refer to the illness representations of the *Common Sense Model of Self-Regulation* (CSM, [7]) as the underlying theoretical framework, which Scott et al. [3] also used in their model.

Help-seeking for mental illness is understood as a process of experiencing symptoms, identifying them as such, forming an intention, and lastly actual help-seeking [7–9]. The identification of symptoms of mental illness is a crucial starting point to forming intention [10–13]. However, a symptom is subjectively not necessarily conceptualised as illness because subjective experience and perceived functional changes influence symptom identification [14]. Therefore, the help-seeking process must start with becoming aware of one’s complaints as a symptom of mental health issues (i.e., self-identification).

Moreover, previous treatment experience influences the help-seeking process, including self-identification and intention [15] by reducing stigmatising attitudes [16]. Stigma in turn has been identified as a major barrier, hindering both self-identification and help-seeking initiation [13]. The types of stigma [17] differ in their influence towards help-seeking [18]. While evidence of the impact on perceived public and anticipated stigma on help-seeking is mixed [19, 20], internalized stigma has consistent negative influences on help-seeking attitudes and intention [13, 20–24]. In order to explain help-seeking for mental health problems, it is important to consider these outlined *superordinate variables* originating from the public and social reactions as well as personal experiences [3]. They might influence present internal beliefs and attitudes (i.e., *intermediary variables*) with direct influences on the *help-seeking process*. Figure 1 presents our *Seeking Mental Health Care Model* [7], which includes hypotheses most interesting for the total model whilst controlling for others. This is especially relevant for the multiple empirical associations between treatment experience and intermediary variables that are mentioned in the following sections. Therefore, instead of formulating every single correlational hypotheses to replicate each empirical finding, we see treatment experiences as superordinate influence that correlates with several model variables, but only formulate specific hypotheses of interest. In the next section we will present the intermediary variables (Fig. 1).

*Continuum beliefs of mental health and illness* are associated with lower stigmatising attitudes towards people with mental illness [25]. Concerning, for example, drinking behaviour, the understanding of drinking along a continuum is currently discussed as a way to promote self-identification as part of a broad operationalisation of problem recognition [26, 27]. Evidence is sparse for continuum beliefs of people with depression [25], however there are indications that a continuum model increases perceived similarities with a depression vignette [28].

*Mental health literacy*, the capacity to access, process, and apply information about mental illness and treatment [29], is recognised as an intermediary variable. It is associated with lower stigma and higher self-identification as well as being positively influenced by treatment experience [13, 15]. However, self-stigma seems to act as a barrier between mental health literacy and help-seeking, therefore these associations need further reassurance [30].

*Causal beliefs* concerning one’s mental health problems are associated with higher self-identification and are influenced by treatment experience [15]. However, there is conflicting evidence on various causal beliefs (e.g., biomedical, childhood related) and their relation to stigma (e.g., [31, 32]). A balanced biopsychosocial model based on biopsychosocial model of health [33] might reflect a realistic and effective causal belief model which influences help-seeking behaviour while balancing possible adverse effects of stigma [34, 35].

*Self-efficacy* [36] for engaging in health behaviours has mainly been investigated for physical health [37, 38]. For mental health help-seeking, it might be important to differentially consider task-specific self-efficacy. On the one hand, *seeking professional help self-efficacy* has been shown to be beneficial for help-seeking [9]. On the other hand, *self-help self-efficacy* might decrease professional help-seeking in favour of tendencies to help oneself [39, 40], however this has rarely been investigated. Concerning the association between *treatment experience* and *self-efficacy* we assume that they are positively associated, because treatment is shown to reduce self-stigma which is in turn negatively associated with self-efficacy [41–43].

Expanding the view to general health-related decision making, the *Common-Sense Model of Self-Regulation* (CSM; [7]) is applied. CSM has been investigated extensively in the context of physical health [44], but has also been replicated for the mental health context [45]. It assumes a dynamic process from becoming aware of health threats, their emotional processing to treatment use through an interplay of illness representations [14]. These include representations of the identity (labelling) and timeline expectations of symptom development, their causes, perceived controllability, coherence with internal concepts of illness, and consequences of past and present illnesses [14, 46]. In a recent meta-analysis, Cannon et al. [45] found large effects of illness representations on treatment use, however, only a few studies investigate treatment use for mental illness resulting in a need for further research in this field [45]. Therefore, we theoretically match the CSM illness representations to the above mentioned empirical variables influencing professional help-seeking behaviour (see Fig. 1). The subjective sense of illness is matched with the *consequence* representation and self-identification of having a mental illness with the *identity* representations. Especially the intermediary variables can roughly be assigned: The extent towards a person’s belief in a continuum of mental health and illness can be understood as the representation *coherence*. Depression literacy includes people’s concept of *illness timeline,* causal beliefs refer to *causes* representation, and self-efficacy represents the aspect of *perceived controllability*. These illness representations develop and change throughout the process of the illness, they interact with illness perceptions, subjective complaints, and experienced emotions. As a consequence, they respectively influence future processing of health threats and an integration of new experiences. Stigma negatively influences the CSM representations, as found for most of the described variables [47].

Our aim is to test the *Seeking Mental Health Care Model*, with the novel approach of incorporating superordinate stigma and experiences and five intermediary internal processes of illness perception that empirically influence help-seeking. Therefore, we use a prospective study on help-seeking in a population sample with currently untreated depressive symptoms [7].

## Methods

This study is part of a project funded by the German Research Foundation, with a published study protocol [7] and preregistration in the German Clinical Trial Register (DRKS00023557). Data was collected by the online panel *respondi AG* via their online platform *Mingle* between January and September 2021. If screened eligible for study participation the panellists were informed about study content and procedure after which they could give electronic informed consent. The study included a quasi-experimental intervention design manipulating the intermediary variables. For the analyses, pre-intervention data is used, except for the behaviour variable which could only be assessed three to six months post intervention. However, this should not be an issue for testing interrelations of the variables, as the study has a fractioned factorial design. Furthermore, this study is not part of the original analysis but a secondary data analysis testing the model as a whole. For more information on the study design and interventional manipulation, refer to the study protocol [7].

### Sample & power analysis

Participants without current professional treatment and with at least mild depressive symptoms were included (PHQ-9 sum score ≥ 8; 48). In total, *N* = 1867 participants completed the questionnaire, which assessed stigmatising attitudes, intermediary variables and help-seeking variables. The final sample size consisted of *N* = 1368, because participants were excluded if they completed the study under half the median duration [48] or had apparent monotone answer profiles (*n* = 116), if their PHQ-9 score was < 8 after the second assessment time 36 hours later (*n* = 362), or due to conflicting information about an individual’s gender between the study points (*n* = 12). Our sample only consisted of *n* = 9 people who reported being of diverse gender. Because of the small case number we decided to exclude them from the analyses, however we emphasize the importance of focusing on hard to reach, yet especially vulnerable gender groups [49], because otherwise this methodologically reasonable exclusion only reproduces discrimination regarding minorities access to care [50]. All participants were contacted for follow-ups after three and six months in which help-seeking behaviour was assessed. Altogether, *N* = 829 participated till the end of the study six months later. Refer to the study protocol for information on participant recruitment and content of assessment [7].

Because the path analysis is done in addition to the analyses reported in the study protocol [7] we recalculated the necessary sample size for this specific analysis. The estimated minimum sample size had to be *N* = 200 (as proposed by Boomsma [51];). With a stricter rule of thumb, with five to ten needed observations per estimated parameter [52, 53], we estimated a required sample size between *N* = 645 to 1290. Therefore, we concluded that our sample size and statistical power is sufficient.

### Measures

All measures used for the path model analysis are listed in Table 1. The operationalisation of help-seeking intention and behaviour as well as other adapted variables are elucidated in further detail below. For extensive descriptions of all measures employed in the study refer to the study protocol [7].

**Table 1:** Overview of the measures used in the current study

Variable	Measurement (items)	Scoring (range)	α	Reference
*Superordinate variables*				
Previous treatment experience^a^	1-item question	0 = no experience; 1 = experience	n.a.	n.a.
Stereotype Agreement	Self-Stigma of Mental Illness Questionnaire, Short-Form (5)	mean score (1–5)	.84	[54]
Stereotype Awareness	Self-Stigma of Mental Illness Questionnaire, Short-Form (5)	mean score (1–5)	.80	[54]
Self-Stigma of Seeking Help	Short-Form of the Self-Stigma for Seeking Help Questionnaire (3)	mean score (1–5)	.92	[55]
*Intermediary variables*				
Continuum Beliefs	Adapted from different instruments (9)	mean score, (1–5)	.68	n.a.
Mental Health Literacy	Depression Literacy Scale (12)	sum score of right answers	.72	[56]
Causal beliefs of mental illness^a^	BioPsychoSocial Causal Model (18)	index score	n.a.	[16, 57]
Self-efficacy to self-help	Adapted items from the BRAHMS study (6)	mean score (1–5)	.80	[58]
Self-efficacy to seek professional help	Adapted items from Healthcare Use Self-Efficacy List (7)	mean score (1–5)	.86	[59]
*Help-Seeking Process variables*				
Sense of illness^a^	Brief Illness Perception Questionnaire (8)	mean score (1–7)	.66	[46]
Self-identification as having a mental illness	SELF-I (5)	mean score (1–5)	.90	[60, 61]
Help-seeking intention^a^	List-wise assessment (15)	maximum score (1–7)	n.a.	[62]
Help-seeking behaviour^a^	List-wise assessment (15)	0 = no; 1 = yes	n.a.	[62]
*Control variables*				
Age	1-item question	≥18, continuous	n.a.	n.a.
Gender	1-item question	1 = female, 2 = male	n.a.	n.a.
Depression severity	Patient Health Questionnaire (9)	sum score (0–27)	.70	[63]

*Ranges*: higher scores indicate higher agreement to variables. α = internal reliability of instruments in the current study sample. *n.a*. Not applicable; for references due to scale development within the current project. ^a^variables and measurement explained in detail in the text

With the *Brief Illness Perception Questionnaire* (B-IPQ; [46]) we assessed the participants subjective sense of illness. Eight items are rated on a 7-point Likert scale. One item assessing causal beliefs was excluded due to its open response format. A mean score was calculated, representing the degree to which the illness is perceived as threatening or benign, which we assume to be an important trigger point for the model. A higher score reflects a more threatening view of the illness. This operationalisation is suggested by Broadbent [64].

*Causal beliefs of mental illness* were assessed with a list of 18 possible causes [57]. Participants were asked to rate whether they believe a cause (e.g., “loneliness”) could be responsible for their experienced complaints. Rating was assessed on a 5-point Likert scale from 1=“definitely is not a cause” to 5=“definitely is a cause”. Internal consistency was acceptable with *α* = .78. With the aim of determining a *balanced biopsychosocial causal model* we calculated the *BPS-CM index*, which represents to what extent five different factors (i.e., biogenetic, psychological, social, environmental, comparable to Stolzenburg et al. [16]) are seen as possible causes for one’s own mental complaints. Between-factor variance is considered. A higher score indicates a more heterogeneous belief system. To determine the index, the 18 items were subsumed into factors and the within-factor agreement as well as the between-factor variance was calculated and multiplied with each other to consider the homogeneity of the belief system. Then, all items were recoded into a binary format: 0 = “definitely is not a cause” and 1 = “definitely is a cause”. The within-factor agreement was determined 0% representing the persons belief that none of the items belonging to one factor are a possible cause whilst 100% represents the belief that all items of one factor are considered to be possible causes (each factor contributing 20% equally). The between-factor variance was calculated to consider the homogeneity of the belief system. Higher inverted variance can be interpreted as higher similarity between factor means. The BPS-CM index ranges from 2.12 to 42.80. The range is specific to the sample, seeing as the variance is used to calculate the index.

*Previous treatment experience* was assessed with the question “Have you ever received treatment for mental illness in your life?”. Multiple responses were possible for: “medical treatment”, “psychotherapy”, “art-, music- and/or sport-therapy”, “self-help groups”, “coaching and counselling”, and “online or telephone therapy”. The answers were collapsed into a dichotomous variable with 0 = “no experience”, 1 = “treatment experience”.

*Intention to seek help from a health professional* was assessed with an 15-item list adapted from Pescosolido and Boyer [62]. Participants rated the probability of seeking help from potential persons (e.g., psychiatrist) and institutions (e.g., counselling centre) on a 7-point Likert scale from 1 = “extremely unlikely” to 7 = “extremely likely”. Internal consistency was good with *α* = .87. To operationalize intention to seek professional help, maximum scores across the items *counselling centre*, *general practitioner*, *psychologists*, *psychotherapists*, *psychiatrists*, and *neurologist* are determined reflecting the diversity of professional help sources. The final choice was based on the results of an explorative factor analysis in which these items loaded best together.

*Help-seeking behaviour* was assessed with the same list as *intention*. Respectively, participants were asked if they sought help in the past 3 months (0 = “no”, 1 = “yes”). When stating “yes”, a subsequent question specified whether they sought help for their psychological complaints (1 = “yes, exclusively”, 2=“yes, amongst other complaints”, 3=“no, because of other complaints). Only if they stated 1 or 2, were their answers coded as seeking help for their depressive complaints. To recode this, we collapsed responses for both follow-ups into dichotomous variables of help-seeking within the last three or six months with 0=“did not seek help” and 1=“sought help during either the three or six month follow-up”. The same persons and institutions described above were used to operationalize professional help-seeking behaviour.

### Statistical analysis

No missing scale values are detected within the relevant data. Total drop-out rate was 28.14% between baseline assessment and both follow-ups together (i.e., participation in either three- or six-month follow up). To examine potential reasons for attrition we conducted logistic regressions in which dropout is defined as not providing any follow-up data, the variable is dummy-coded as 0 = “no missing variable” and 1 = “missing value due to drop-out” [65]. Predictors were self-identification, general health condition, depression severity, treatment experience, and various sociodemographic variables chosen broadly to accurately analyse possible reasons for attrition. We report Odds Ratios (*OR*) and 95% confidence intervals (*CI*) for the significant coefficients.

To assess the proposed influences as shown in the *Seeking Mental Health Care Model* (Fig. 1), we conducted a path model analysis [66–68]. To check the hypothesised assumptions concerning the associations between the respective variables we performed Pearson’s product-moment and Kendal-Tau correlation analysis. We then conducted the path model analysis with the *lavaan package* [69] using the statistics software *R version 4.0.3* [70]. The algorithm for the path model correlation matrix, exact specifications, and fitting commands, i.e., WLSMV estimator and pairwise deletion of missing values (refer to [71]), can be seen in the supplement (Table S1). We controlled for depressive complaints (PHQ sum score), age, and gender (dummy variable, “female” is the reference category), as well as for exogenous variables without specific hypotheses. All continuous variables were *z*-standardised. Standardised path estimates (interpretable as partial β-coefficients) for the different paths as well as corrected *R*^*2*^ estimates for the respective explained variances for the endogenous variables are reported. The usual model fit indices Χ^2^ (df and *p*-value), RMSEA, CFI, NFI, SRMR, and AGFI are reported.

## Results

### Sample characteristics

The final sample size consisted of *N* = 1368, of which 65.6% identified as female and the mean age was 42.38 (*SD* = 15.22). The mean depression score was 12.50 (*SD* = 4.08), indicating that the severity of depressive symptoms ranged mainly from mild to moderate [72]. People graduated school after 12/13 years (52.4%), 10 years (34.1%), or 9 years of schooling (11.3%) and some were still in school or had not graduated (2.2.%). 49.2% had professional training or finished an apprenticeship, and 22.7% had a university degree (i.e., bachelor’s degree = 7.8%, master’s degree = 13.9%, PhD = 1.0%). Collapsed sample size after 6 months was *N* = 983. Attrition analysis revealed that dropout was more likely if the participants were younger (*OR* = 0.963; *CI* = 0.952, 0.974), came from bigger households (*OR* = 1.174; *CI* = 1.047, 1.317), and if they had nine compared to 12 years of schooling (*OR* = 1.788; *CI* = 1.141, 2.803) while 11 other variables had no significant influence on dropout.

### Path model analysis: the Seeking Mental Health Care model

The associations between the superordinate, intermediary, and help-seeking process variables are presented in Table 2.

**Table 2:** Correlation matrix for all superordinate, intermediary, and help-seeking process variables

	1	2	3	4	5	6	7	8	9	10	11	12	13	14	15	16
1. Age	–															
2. Gender	.20^**^	–														
3. Depression severity^a^	−.10^**^	−.02	–													
*Help-seeking variables*																
4. Subjective sense of illness^b^	.14^**^	.07^**^	.40^**^	–												
5. Self-identification^c^	−.06^*^	−.13^**^	.34^**^	.40^**^	–											
6. Intention	.19^**^	.05^*^	.07^*^	.12^**^	.14^**^	–										
7. Behaviour	.13^**^	.03	.09^**^	.11^**^	.11^**^	.36^**^	–									
*Intermediary variables*																
8. Continuum beliefs	−.13^**^	−.12^**^	−.09^**^	−.09^**^	.13^**^	−.06^*^	−.00	–								
9. Depression literacy^d^	−.04	−.11^**^	.06^*^	.06^*^	.32^**^	−.02	.03	.35^**^	–							
10. Causal beliefs^e^	−.05^*^	−.02	.09^**^	.03	.18^**^	.09^**^	−.01	−.12^**^	−.04	–						
11. Self-efficacy to self-help	.02	.03	−.06^*^	−.20^**^	−.22^**^	.15^**^	.05^*^	−.02	−.10^**^	−.09^**^	–					
12. Self-efficacy to seek-help	.01	.07^**^	−.19^**^	−.29^**^	−.27^**^	.21^**^	.07^**^	.02	−.07^**^	−.12^**^	.42^**^	–				
*sSuperordinate variables*																
13. Stereotype awareness^f^	−.18^**^	−.01	.16^**^	.07^*^	.12^**^	−.06^*^	−.05	.01	.03	.07^**^	−.07^**^	−.09^**^	–			
14. Stereotype agreement^g^	−.07^**^	.08^**^	.04	.01	−.19^**^	−.02	−.08^**^	−.30^**^	−.32^**^	.18^**^	−.02	−.05	.30^**^	–		
15. Self-stigma of help seeking^h^	−.02	.11^**^	.09^**^	.08^**^	−.19^**^	−.26^**^	−.14^**^	−.22^**^	−.22^**^	.06^*^	−.16^**^	−.22^**^	.08^**^	.33^**^	–	
16. Treatment experience	.12^**^	−.06^*^	.10^**^	.14^**^	.37^**^	.18^**^	.19^**^	.08^**^	.24^**^	.13^**^	−.05^*^	−.02	.00	−.11^**^	−.24^**^	–

Pearson & Kendal-Tau correlation coefficients. * *p* < .05, ** *p* < .01

^a^Patient Health Questionnaire (Depression Sub-Scale) to measure depression severity

^b^Brief Illness Perception Questionnaire

^c^Self-identification as having a mental illness Scale

^d^Depression Literacy Scale

^e^Causal Belief Index representing a balanced BioPsychoSocial Causal Model

^f^Short-Form of the Self-Stigma of Mental Illness Questionnaire – Public

^g^Short-Form of the Self-Stigma of Mental Illness Questionnaire – Self

^h^Short-Form of the Self-Stigma for Seeking Help Questionnaire

**Fig. 2:**
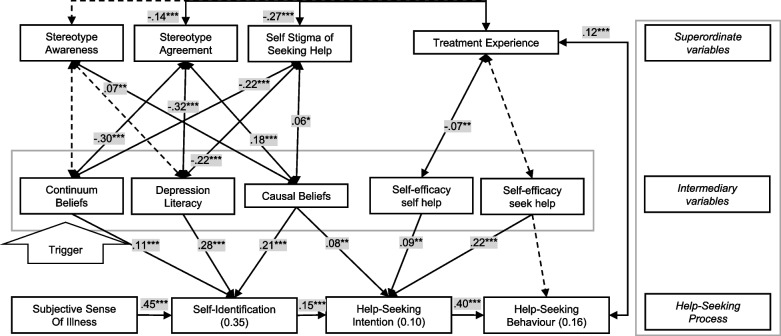
Path model for seeking mental health care from a professional source. (Note: *N* = 1368. Standardized estimates with solid lines indicating significant relationships at *p < 0.05, **p < 0.01, ***p < 0.001; broken lines non-significant, hypothesised relationships. Double headed arrows represent associations between the variables. One headed arrows represent predictions on endogenous variables. In brackets the estimated R^2^ for the endogenous variables. Modell is controlled for depression severity, age, and gender)

Figure 2 shows the path model for seeking mental health care from a professional source (i.e., general practitioner, psychologist, therapist, psychiatrist, neurologist, or counselling centre). Significant standardised path estimates and non-significant yet hypothesised associations are reported. In the supplementary material we report the full model with all coefficients predicting or associated with the endogenous variables (Table S2).

The model confirmed most of the proposed predictions of the help-seeking process and the associations between the superordinate and intermediary variables except for the prediction of previous treatment experience on self-efficacy to seek help and the prediction of self-efficacy to seek help on help-seeking behaviour. Overall, the model explained 35% of the variance of self-identification, 10% of intention to seek help and 16% of the variance of seeking a health-care professional after either three or six months. Model fit was excellent with *Χ*^*2*^ = 24.968, *df* = 7, *p* < .001, RMSEA = 0.059, CFI = 0.989, NFI = 0.988, SRMR = 0.012, and AGFI = 0.999.

## Discussion

The aim of this study was to test a comprehensive *Seeking Mental Health Care Model* in a sample of people with currently untreated depressive symptoms. The model contains key internal influencing variables, from superordinate over intermediary to help-seeking process variables, to explain professional help-seeking behaviour due to mental health problems. A path analysis confirmed the general structure of the model with an excellent model fit, significantly predicting help-seeking behaviour while revealing reciprocal influences between process variables. Therefore, we can conclude that there are interdependent attitudinal and cognitive variables determining how symptoms are identified and processed in order to seek professional help. They support a theoretical proposed process comparable to processing according to CSM.

The *subjective sense of illness* is the starting point of the model triggering further intra-psychological variables and representing the dynamic nature of the model with subsequent variables relying on preceding processes. We operationalised the B-IPQ-R variables using the mean score, according to Broadbent [64], even if this is less subtle then the CSM would suggest [14]. When the illness is subjectively perceived as more threatening, the mean *self-identification as having mental illness* increased, which is in line with the work from O’Mahen et al. [73]. As expected, self-identification was higher the more heterogeneous the causal belief system and the higher depression literacy, which is in line with our previous findings [13, 15]. Further, on average, participants were more likely to recognize their current complaints as signs of mental illness if they report higher belief in a continuum of mental health and illness. To our knowledge, this is an addition to previous findings, which were rather focused on enhancing problem recognition for at-risk drinking through continuum beliefs [27]. The direct link between continuum beliefs and self-identification of having a mental illness should be investigated further (e.g., [74]).

For the prediction of *help-seeking intention* all hypothesized paths were significant. Higher self-identification lead to higher help-seeking intention meaning that identifying one’s own complaints as signs of a mental illness is an antecedent of intention to seek help [11–13]. Self-efficacy for professional help had the strongest influence on intention. The belief in a balanced causal model [35] as well as self-efficacy to help oneself had positive but small (ß < .10) influences. We did not assume the positive effect of self-help self-efficacy on intention, as this self-efficacy implies a desire to help oneself found to reduce the intention to seek professional help [75]. However, the moderate correlation between the two forms of self-efficacy supports that forming an intention to search for help could also be partly interpreted as an act of self-help. Self-help should therefore not solely be understood as having to deal with problems completely by oneself but rather as a set of behaviours engaged towards strengthening one’s health and self-care [76]. In contrast to other studies [8, 9], help-seeking intention was predicted with less explained variance, probably due to the lack of the well-established predictor *help-seeking attitudes* which was omitted because we aimed to examine less established variables.

For *help-seeking behaviour*, we found a well-established pattern of results. The influences of most variables were mediated through help-seeking intention as the strongest predictor [9, 13]. Along with that, our hypothesized influence of self-efficacy for professional help against our expectation was not significant, once again speaking for indirect effects of internal variables on behaviour via intention. Furthermore, having had treatment experience increased the probability to seek help, which is in line with other findings [13, 77, 78]. Our path model was able to explain 16% of variance of help-seeking behaviour. Keeping in mind that the strength of our study is that we used an outcome of actual utilisation of professional care within 6 months, uncontrolled external influences are probable. Moreover, the investigation of structural barriers was outside of our research scope, although their influences on help-seeking behaviour are evident as well [5, 79]. It should be noted that the resulting healthcare use might be influenced by the Covid-19 pandemic which was shown to overall decrease help-seeking behaviour for mental health [80]. Reinforced gender differences in help-seeking during Covid-19 were found for young people [81], which points to important future research directions. Moreover, sources of treatment like virtual treatments or other related (non-mental health) professionals like priests, teachers, police etc. should be investigated. This was outside our scope but could be differently affected by the influencing variables analysed in this study.

Regarding superordinate variables, t*reatment experience* was significantly associated with *stigma* with small to moderate coefficients, which is in line with other research [16, 82]. The proposed associations between treatment experience and self-efficacy to seek help was not significant. Treatment experience per se might not influence the self-efficacy to seek help for subsequent health care utilisation. However, we didn’t include quality of treatment experience although it might be worthwhile to further investigate how quality of treatment experience actually affects stigma [83] and how specific experiences can be highlighted in interventions. Furthermore, it might be interesting to investigate how treatment experience and self-efficacy might both contribute to another construct, e.g., disease self-management [84], or are moderated, e.g., through trust towards healthcare providers [85]. Also, multiple associations of treatment experience with other variables were controlled for as to not overload the scope of this work.

We hypothesised associations between *stigma* and *intermediary variables*, i.e., continuum beliefs, mental health literacy, and causal beliefs. Lowest and mostly non-significant influences emerged with stereotype awareness replicating findings of its low influence on intrapsychic help-seeking processes [78]. We found a significant negative correlation of β = −.30 for stereotype agreement and β = −.22 for self-stigma of seeking help with continuum beliefs, which is in line with the overall negative associations found in public samples [25]. There is an absence of comparable samples, except for the work by Thibodeau et al. [86] who reported non-significant results. Because our results refer to a sample of people reporting depressive symptoms themselves, these results are an addition to existing literature, showing a tendency towards the effectiveness of continuum messages. Similarly, directed associations could be found for stigma and depression literacy opposing findings that stigma isn’t influenced by depression literacy [87]. For causal beliefs, we opted for a balanced biopsychosocial model, which we operationalised integrating the homogeneity of the belief system across different causal factors. Our aim was to balance the stigmatising potential of different causal beliefs [16, 57]. Still, our results indicate that more heterogeneous consideration of types of causes is associated with higher stigma. Due to these consistent findings across different operationalisations, future studies should use caution when incorporating causal belief messages as part of psychoeducative public health campaigns that aim to reduce stigma on an individual level. Importantly, we leave investigations of people with clinical diagnoses to future studies [4], since we wanted to include a population without current treatment.

### Limitations

Our analyses are based exclusively on self-reported data. New instruments yet lacking full psychometric validation were used for continuum beliefs, causal beliefs, self-efficacy measures and treatment experience. However, the new questionnaires are based on existing validated items and therefore should still be interpretable, yet not fully comparable to other studies and thus need validation. We opted for a broad operationalisation of help-seeking including general practitioners, counselling centres, or neurologists, additional to psychotherapeutic or psychiatric professionals. We aimed to reflect help-seeking with high external validity since, for example, nearly three of four people with depression are treated by general practitioners [88]. Future studies could, on the one hand, investigate single health care providers to understand specific paths to health care, and on the other hand, incorporate informal sources as a mediating first step towards professional health care use [89].

Additionally, limitations to the representativeness of the sample are raised. On the one hand, we investigated a sample in Germany, limiting generalisability to other countries, partly due to differences regarding healthcare accessibility and statutory health insurance. Moreover, we limited our analyses to intrapersonal processes and their influencing factors. It would be important to replicate our analyses with focus on structural barriers, including factors of perceived and actual costs, availability and accessibility (e.g., transportation, hours of work, childcare requirements). Furthermore, even though we controlled for the influence of age, gender, and depression severity, there might be an influence of the socioeconomic status [79], including education, occupation and income. We did not include these control variables, due to the models already extensive inclusion of multiple variables and our aim of analysing internal processes of help-seeking. An interplay between internal and external factors could be a beneficial adaptation of the model, though the complexity might be an issue when statistically analysing such a comprehensive model.

The study is likely to contain a self-selection bias, as only motivated people with computer skills and access to the online panel might have been interested and able to participate. This longitudinal study assessed help-seeking behaviour over a period of up to three to six months with a drop-out from baseline to follow-ups. Attrition analyses have shown that those who drop out systematically differ from the rest of the sample in certain characteristics, further limiting representativeness. Additionally, this attrition is an indication of variables to address in further research, such as household size. So far, we limited our analyses to direct influences of the intermediary variables to provide insight on the influences of direct processes not considering combined influences nor possible reciprocal interactions between the intermediary variables. Correlational analyses revealed that they are partly negatively associated, suggesting that further analyses on the most effective combinations are needed, e.g., for drawing in-depth practical implications for creating help-seeking interventions with combined psychoeducational content.

## Conclusion

In summary, we were able to replicate findings of various influences on the help-seeking process expanding existing research of help-seeking variables and stigma through an intermediary level of subjective illness representations according to the *Common-Sense Model of Self-Regulation* [14]. We examined a comprehensive approach and focused on internal attitudes of people currently not receiving treatment. Further studies should allow for an interaction between intermediary variables and expand the model to include both internal and external factors, as well as reciprocal associations. Although results need assurance in a sample with diagnosed depression, we see clinical implications of the discussed findings. Strengthening intermediary variables to decrease stigmatising attitudes and increase help-seeking behaviour in an interventional setting needs to be done with a comprehensive and holistic understanding of such a multifactorial model, for example by focusing on a destigmatising way to increase the likelihood that people with ill-health self-identify as having mental health symptoms. In sum, the *Seeking Mental Health Care Model* provides an empirically validated framework to consider the help-seeking process of people with untreated depressive symptoms within a widened approach considering many internal variables.

## Supplementary Information

**Additional file 1: Table S1.** R syntax and fitting commands for the path model analysis for the Seeking Mental Health Care Model. **Table S2.** Identified path model for seeking mental health care from a professional source.

## Data Availability

The datasets generated during the current study are available from the corresponding author on reasonable request.

## Abbreviations

B-IPQ: Brief Illness Perception Questionnaire
BRAHMS: Berlin Risk Appraisal and Health Motivation Study, 1996
BPS-CM: Biopsychosocial Causal belief Model index
CSM: Common Sense Model of Self-Regulation
DFG: Deutsche Forschungsgemeinschaft (i.e., German Research Foundation)
PHQ-9: Patient Health Questionnaire (Depression Sub-Scale)
SELF-I: Self-identification as having a mental illness
SSMI-public & -self-SF: Short-Form of the Self-Stigma of Mental Illness Questionnaire
SSOSH-SF: Short-Form of the Self-Stigma for Seeking Help Questionnaire
