# Supplementary material for: The Seeking Mental Health Care model: prediction of help-seeking for depressive symptoms by stigma and mental illness representations
Source: BMC Public Health. 2023 Jan 10;23:69. doi: 10.1186/s12889-022-14937-5 (PMC9831378; doi:10.1186/s12889-022-14937-5)
Supplement: Supplementary file 1 — Additional file 1: Table S1. R syntax and fitting commands for the path model analysis for the Seeking Mental Health Care Model. Table S2. Identified path model for seeking mental health care from a professional source. [file 12889_2022_14937_MOESM1_ESM.docx]

Supplementary Material

**The Seeking Mental Health Care Model: Prediction of help-seeking for depressive symptoms by stigma and mental illness representations**

# Content

In this supplement we report the *R syntax script* with specifications and fitting commands (S1) as well as the *full model* with all coefficients predicting or associated with the endogenous variables (S2).

| **Table S1.**  R syntax and fitting commands for the path model analysis for the Seeking Mental Health Care Model. |
| --- |
| #packages: "lavaan"& "lavaanPlot"  pathmodel <- '  #equations where endogenous variables are predicted by respective exogenous variables  Zselfi ~ Zipqr + Zcontinuum + Zdlit + Zcausal  Zintention ~ Zselfi + Zcausal + Zselfhelp + Zseekhelp  behav ~ Zintention_all + Zseekhelp  #equations estimating covariances between endogenous and exogenous variables, as theoretically plausible (including control variables)  Zselfi ~~ exp + Zssmi_pub + Zssmi_self + Zssosh + Zphq + Zage + dummy_female  Zintention ~~ exp + Zssmi_pub + Zssmi_self + Zssosh + Zipqr + Zcontinuum + Zdlit + Zphq + Zage + dummy_female  behav ~~ exp + Zssmi_pub + Zssmi_self + Zssosh + Zipqr + Zphq + Zage + dummy_female  #equations estimating all possible covariances between exogenous variables (including control variables)  Zssmi_pub ~~ Zssmi_self + Zssosh + exp + Zipqr + Zcontinuum + Zdlit + Zcausal + Zselfhelp + Zseekhelp + Zphq + Zage + dummy_female  Zssmi_self ~~ Zssosh + exp + Zipqr + Zcontinuum + Zdlit + Zcausal + Zselfhelp + Zseekhelp + Zphq + Zage + dummy_female  Zssosh ~~ exp + Zipqr + Zcontinuum + Zdlit + Zcausal + Zselfhelp + Zseekhelp + Zphq + Zage + dummy_female  exp ~~ Zipqr + Zcontinuum + Zdlit + Zcausal + Zselfhelp + Zseekhelp + Zphq + Zage + dummy_female  Zipqr ~~ Zcontinuum + Zdlit + Zcausal + Zselfhelp + Zseekhelp + Zphq + Zage + dummy_female  Zcontinuum ~~ Zdlit + Zcausal + Zselfhelp + Zseekhelp + Zphq + Zage + dummy_female  Zdlit ~~ Zcausal + Zselfhelp + Zseekhelp + Zphq + Zage + dummy_female  Zcausal ~~ Zselfhelp + Zseekhelp + Zphq + Zage + dummy_female  Zselfhelp ~~ Zseekhelp + Zphq + Zage + dummy_female  Zseekhelp ~~ Zphq + Zage + dummy_female  Zphq ~~ Zage + dummy_female  Zage ~~ dummy_female  #estimating the variances of the exogenous variables (including control variables)  Zssmi_pub ~~ Zssmi_pub  Zssmi_self ~~ Zssmi_self  Zssosh ~~ Zssosh  exp ~~ exp  Zipqr ~~ Zipqr  Zcontinuum ~~ Zcontinuum  Zdlit ~~ Zdlit  Zcausal ~~ Zcausal  Zselfhelp ~~ Zselfhelp  Zseekhelp ~~ Zseekhelp  Zphq ~~ Zphq  Zage ~~ Zage  dummy_female~~ dummy_female  #estimating the residual variances of the endogenous variables  behav ~~ behav  Zintention ~~ Zintention  Zselfi ~~ Zselfi  '  #the fitting command for the model  fit <- lavaan(pathmodel, estimator = "WLSMV",  missing = "pairwise",  test = "robust",  data = data)  options(max.print = 10000)  summary(fit, fit.measures = TRUE, standardized = TRUE, rsquare = TRUE)  standardizedSolution(fit)  fitted(fit)  coef(fit)  fitMeasures(fit)  #quick and dirty way to visualise the main results  lavaanPlot(model = fit, node_options = list(shape = "box", fontname = "ArialNarrow"),  edge_options = list(color = "black"), coefs = TRUE, stand = TRUE,  covs = FALSE,stars = c("regress", "covs"), sig = .05) |

| **Table S2.**  Identified path model for seeking mental health care from a professional source | | | | | | |
| --- | --- | --- | --- | --- | --- | --- |
|  | self-identification as having a mental illness | | intention to seek help | | help-seeking behaviour | |
|  | path estimation | *95% CI* | path estimation | *95% CI* | path estimation | *95% CI* |
| subjective sense of illness | .45*** | .40; .50 | .15*** | .08; .21 | .10*** | .06; .15 |
| self-identification | – | – | .15*** | .08; .22 | .05* | .00; .11 |
| intention to seek help | – | – | – | – | .40*** | .36; .44 |
| continuum beliefs | .11*** | .06; .16 | -.06* | -.12; -.01 | – | – |
| depression literacy | .28*** | .23; .33 | -.02 | -.08; .04 | – | – |
| causal beliefs | .21*** | .16; .25 | .09** | .03; .13 | – | – |
| self-efficacy to self-help | – | – | .09** | .03; .15 | – | – |
| self-efficacy to seek-help | – | – | .22*** | .16; .28 | .00 | -.04; .05 |
| stereotype awareness | .08** | .02; .13 | -.06* | -.12; -.01 | -.04 | -.08; .01 |
| stereotype agreement | -.14*** | -.19; -.08 | -.01 | -.05; .06 | -.09*** | -.14; -.04 |
| self-stigma of help seeking | -.19*** | -.25; -.14 | -.18*** | -.23; -.13 | -.06** | -.10; -.02 |
| treatment experience (yes)^a^ | .29*** | .25; .34 | .15*** | .10; .20 | .12*** | .08; .16 |
| depression severity | .15*** | .10; .20 | .06* | .01; .11 | .10*** | .06; .15 |
| age | -.11*** | -.17; -.06 | .21*** | .16; .26 | .09*** | .04; .13 |
| gender (female)^a^ | .17*** | .11; .22 | -.06* | -.11; -.01 | -.01 | -.06; .03 |
| *explained variance* | *.35* | | *.10* | | *.16* | |
| **Note.** Number of observations = 1368. Number of model parameters = 129. Path estimations can be interpreted as standardized partial β coefficients; 95% *C*I = 95% confidence interval with lower and upper limit. The explained variance is interpretable as R^2^.  *Χ^2^* = 24.968, *df* = 7, *p* = 0.000; RMSEA = 0.059, CFI = 0.989, NFI = 0.988, SRMR = 0.012, AGFI = 0.999  ^a^ reference category in brackets.  * *p* < .05, ** *p* < .01, *** *p* < .001 | | | | | | |
